# ^68^Ga-Pentixafor PET/CT May Fail to Detect Recurrent Multiple Myeloma with Extramedullary Disease

**DOI:** 10.3390/diagnostics13050871

**Published:** 2023-02-24

**Authors:** Qingqing Pan, Yaping Luo, Xinxin Cao, Jian Li, Fang Li

**Affiliations:** 1 Department of Nuclear Medicine, Chinese Academy of Medical Sciences and Peking Union Medical College Hospital, Beijing 100730, China; 2 Beijing Key Laboratory of Molecular Targeted Diagnosis and Therapy in Nuclear Medicine, Beijing 100730, China; 3 Department of Hematology, Chinese Academy of Medical Sciences and Peking Union Medical College Hospital, Beijing 100730, China

**Keywords:** multiple myeloma, extramedullary disease, ^68^Ga-Pentixafor, PET/CT

## Abstract

Two patients with a history of multiple myeloma experienced a recurrence of the disease.^18^F-FDG PET/CT revealed prominent extramedullary disease as well as multi-foci in the bone marrow, both with increased FDG uptake. However, on ^68^Ga-Pentixafor PET/CT, all the myeloma lesions showed significantly lower tracer uptake in comparison with ^18^F-FDG PET. This false-negative result of recurrent multiple myeloma with extramedullary disease may be a potential limitation of ^68^Ga-Pentixafor in assessing multiple myeloma.

**Figure 1:**
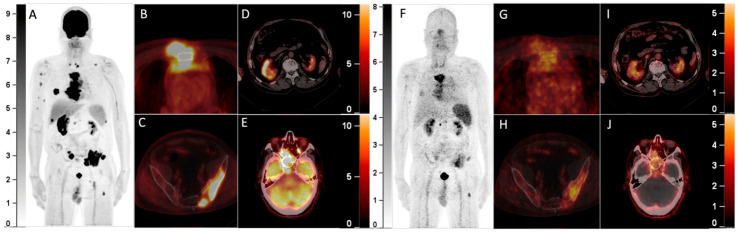
A 64-year-old man with a history of multiple myeloma (MM) for 8 years, recently presented with a parasternal mass and blindness of the right eye. Serum protein electrophoresis and immunofixation electrophoresis showed positivity for the monoclonal protein (16.2 g/L, IgA-λ). A biopsy of the parasternal mass confirmed plasmacytoma. Considering the recurrence of MM, ^18^F-FDG PET/CT was referred. The maximum intensity projection (MIP) of the PET (**A**) detected multi-foci with intense radioactivity all over the body. The axial fusion images (**B**,**C**, bone window) showed most of the FDG-avid foci were located in bone marrow, most prominently in the sternum and pelvis (SUVmax 34.0), accompanied by lytic bone destruction and paramedullary masses. Additionally, the axial fusion images (**D**,**E**, soft tissue window) demonstrated extramedullary disease (EMD) with intense FDG uptake in the right kidney and paranasal sinus (SUVmax 22.3), which also involved the right orbit and temporal lobe. Since ^68^Ga-Pentixafor has been reported to be advantageous over ^18^F-FDG in assessing MM^1,2^, he was included in the clinical trial of ^68^Ga-Pentixafor (NCT03436342). In the MIP (**F**) and corresponding axial fusion images of ^68^Ga-Pentixafor PET (**G**–**J**), the above hypermetabolic foci showed significantly lower tracer uptake as compared with ^18^F-FDG PET (bone marrow lesions: SUVmax 16.5; EMD: SUVmax 7.4).

**Figure 2:**
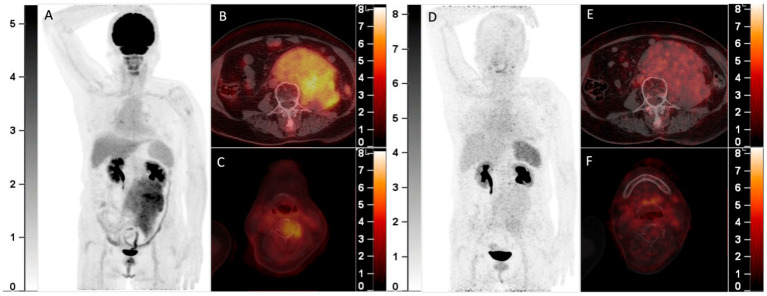
A 70-year-old woman with smoldering MM was found to have a solitary plasmacytoma in the frontal bone that was surgically resected one year ago. Recently, she complained of backache and was found with a retroperitoneal mass. Elevation of the monoclonal protein (19.0 g/L) and the presence of IgA-λ in serum immunofixation electrophoresis and infiltration of plasma cells (17.5%) in bone marrow aspiration confirmed the recurrence of MM. ^18^F-FDG PET/CT was then performed. The MIP image (**A**) showed an FDG-avid mass in the abdomen. The axial fusion images (**B**,**C**) showed the mass was located in the retroperitoneum with uneven FDG distribution (**B**, SUVmax 5.7). Furthermore, several bone marrow lesions with increased FDG uptake and lytic bone destruction were found in the occipital bone, C4 vertebra, and right 8th rib (**C**, SUVmax 4.1). She was also included in the clinical trial and underwent ^68^Ga-Pentixafor PET/CT (**D**, MIP image; **E**,**F**, axial fusion images). However, the retroperitoneal mass and the bone marrow lesions did not demonstrate increased uptake of ^68^Ga-Pentixafor. She then received chemotherapy against MM, and the retroperitoneal mass disappeared after 2 cycles of chemotherapy. ^68^Ga-Pentixafor, a CXCR4-targeted agent, has recently been introduced in MM [1,2,3,4]. Our recent study demonstrated ^68^Ga-Pentixafor had a significantly higher sensitivity than ^18^F-FDG in detecting newly diagnosed MM^2^. However, ^68^Ga-Pentixafor was inferior to ^18^F-FDG in the current two cases of recurrent MM with extensive EMD. CXCR4 is overexpressed in myeloma cells and is responsible for plasma cells’ homing to the bone marrow niche [5,6]. Development of EMD in MM is associated with CXCR4/CXCL12 downregulation through cell adhesion disruption [7,8,9]. In line with the current cases, Lapa C. et al.’s study found that some EMDs were exclusively identified by ^18^F-FDG and were not sensitive to ^68^Ga-Pentixafor [1]. Thus, the significantly lower uptake than ^18^F-FDG of recurrent MM with EMD may be a potential limitation of ^68^Ga-Pentixafor in assessing MM.

## Data Availability

No new data were created or analyzed in this study. Data sharing is not applicable to this article.
